# Discovery of a Novel Antiepileptic Bicyclic Norditerpenoid from the Marine Algae-Derived Fungus *Chloridium* sp. NBU3282

**DOI:** 10.3390/md24100369

**Published:** 2026-10-21

**Authors:** Pengcheng Zhou, Yuqi Su, Yujia Li, Wenhui Zhang, Lixin Chen, Yufeng Xie, Wei Yuan, Fangjian Feng, Lijian Ding, Zhengquan Wang

**Affiliations:** 1 Department of Emergency Medicine, The Affiliated Yangming Hospital of Ningbo University (Yuyao People’s Hospital), Ningbo University, Ningbo 315400, China; 2 Health Science Center, School of Pharmacy, Ningbo University, Ningbo 315211, China; 3 Jiangxi Provincial Key Laboratory of Synthetic Pharmaceutical Chemistry, School of Geography and Environmental Engineering, Gannan Normal University, Ganzhou 314003, China; 4 School of Pharmacy, Changsha Medical University, Changsha 410219, China

**Keywords:** antiepileptic potential, marine algae-derived fungus, norditerpenoid

## Abstract

Two antiepileptic metabolites, nordichloridene (**1**) and 9-chloro-1,2,3,4-tetrahydroacridine (**2**), were isolated from the marine algae-derived fungus *Chloridium* sp. NBU3282. Nordichloridene (**1**) is a new norditerpenoid featuring a bicyclic 6/6 trans-decalin skeleton conjugated with an α,β-unsaturated methyl vinyl ketone side chain. Its structure was elucidated via comprehensive spectroscopic analysis, and the stereochemical configuration was established by NOESY spectroscopy combined with quantum chemical calculations. Compound **2**, previously known only in synthetic chemistry, was identified from a natural source for the first time. Both compounds suppressed pentylenetetrazole-induced hyperactivity in a zebrafish epilepsy model, demonstrating antiepileptic potential via acetylcholinesterase activation and the modulation of epilepsy-associated genes.

## 1. Introduction

Epilepsy is a prevalent chronic neurological disorder affecting over 70 million individuals worldwide, spanning all age groups [1]. It is characterized by recurrent, spontaneous seizures that impose severe psychological and physical burdens [2,3]. Despite remarkable progress in the development of antiepileptic drugs (AEDs), approximately 30% of patients remain refractory to treatment due to therapeutic resistance [4,5]. Mechanisms of multidrug resistance, such as the overexpression of efflux proteins at the blood−brain barrier, further hinder the efficacy of AEDs [6,7]. Additionally, current medications are often associated with adverse side effects [8,9,10]. These challenges emphasize the urgent need for novel agents that are not only more effective, but also safer, particularly for patients with drug-resistant epilepsy.

**Figure 1:**
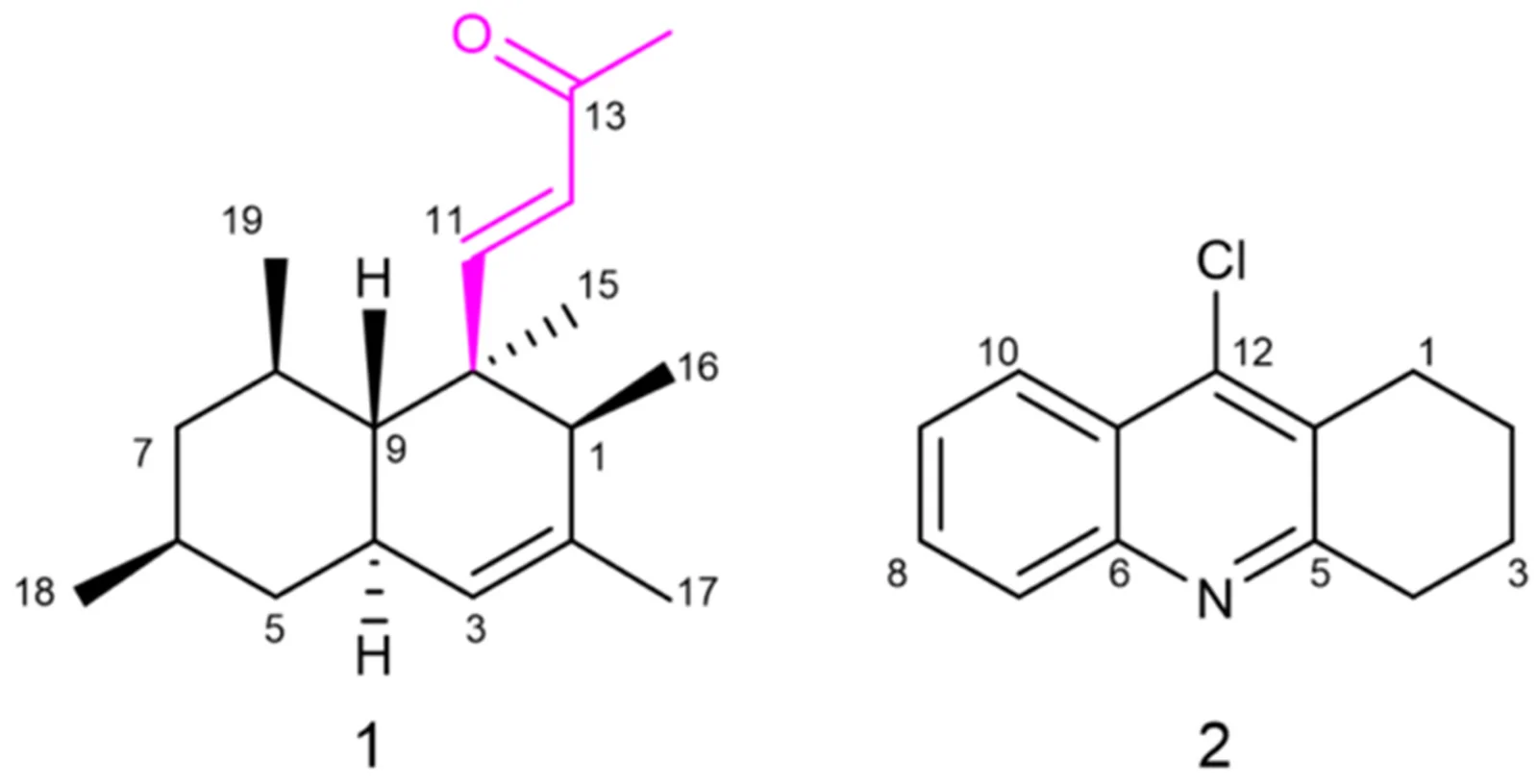
Chemical structures of compounds **1** and **2**.

Marine-derived microorganisms have demonstrated encouraging potential for biosynthesizing unique metabolites with therapeutic effects against neurological disorders [11,12]. *Chloridium*, a fungal genus, is known for its prolific production of a wide range of bioactive metabolites, including potent antibiotics and enzyme inhibitors [13,14]. However, its full pharmacological potential remains largely unexploited in central nervous system (CNS) drug discovery. Our bioactivity-guided investigation of the marine algae-derived fungus *Chloridium* sp. NBU3282 led to the discovery of two antiepileptic metabolites (Figure 1): nordichloridene (**1**), a new norditerpenoid featuring a bicyclic 6/6 trans-decalin core conjugated with an α,β-unsaturated methyl vinyl ketone moiety, and 9-chloro-1,2,3,4-tetrahydroacridine (**2**), previously reported solely as a synthetic compound but now isolated for the first time from nature.

Reported herein are the isolation, structural elucidation, and biological activities of these two compounds, together with a stereochemical study of compound **1**, highlighting their potential as anti-epileptic candidates.

## 2. Results and Discussion

### 2.1. Structure Elucidation

Nordichloridene (**1**) was obtained as a white amorphous powder. The HRESIMS analysis of **1** showed the [M + H^+^] ion at *m*/*z* 275.2376 (calcd for C_19_H_31_O, 275.2375), establishing a molecular formula of C_19_H_30_O with five degrees of unsaturation. The ^1^H NMR data (Table 1) exhibited three olefinic protons: a broad singlet at *δ*_H_ 5.07 (H-3), and two *trans*-coupled doublets at *δ*_H_ 7.15 (d, *J* = 16.6 Hz, H-11) and 5.97 (d, *J* = 16.6 Hz, H-12), characteristic of a conjugated *E*-configured double bond. Three methyl groups resonated as doublets at *δ*_H_ 0.94 (d, *J* = 7.0 Hz, H_3_-16), 0.85 (d, *J* = 6.5 Hz, H_3_-18), and 0.80 (d, *J* = 6.8 Hz, H_3_-19), while three singlet methyl groups appeared at *δ*_H_ 2.21 (s, H_3_-14), 1.01 (s, H_3_-15), and 1.61 (s, H_3_-17). In addition, the ^13^C NMR and DEPT-135 spectra confirmed 19 carbons, including two methylenes (*δ*_C_ 42.1, 46.9), five methines (*δ*_C_ 32.5, 34.8, 39.6, 47.3, 49.2), a saturated quaternary carbon (*δ*_C_ 41.3), an unsaturated quaternary carbon (*δ*_C_ 135.3, C-2), and a conjugated carbonyl carbon (*δ*_C_ 198.0, C-13). These functionalities accounted for three degrees of unsaturation, necessitating two rings to satisfy the molecular formula. Comprehensive analysis of the above data combined with spectroscopic evidence (UV: *λ*_max_ 212 nm; IR: *ν*_max_ 1705 cm^−1^) corroborated the presence of an α,β-unsaturated ketone moiety. Collective interpretation of the HRESIMS, 1D NMR, and spectroscopic data unambiguously established the bicyclic carbon skeleton of **1**, featuring a conjugated enone system.

**Figure 2:**
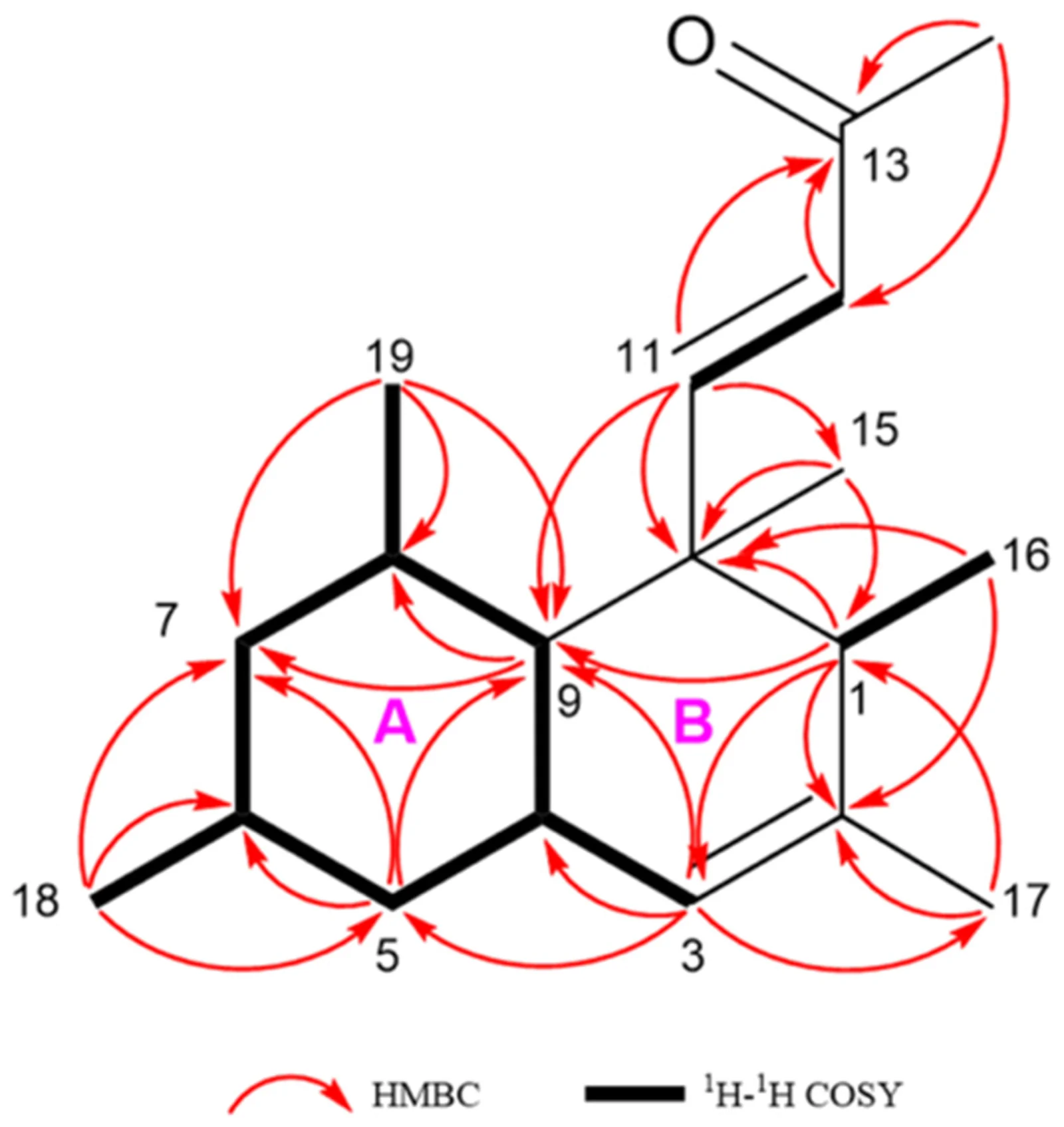
Key COSY and HMBC correlations of **1**.

The planar structure of **1** was determined through a thorough analysis of 2D NMR spectra (Figure 2). Systematic examination of COSY signal clusters within the *δ*_H_ 0.7–1.9 ppm revealed a contiguous spin system corresponding to the CH (4)–CH_2_ (5)–CH (6)–CH_2_ (7)–CH (8)–CH (9)–CH (4) sequence. Key HMBC correlations from H_3_-18 to C-5/C-6/C-7, as well as from H_3_-19 to C-7/C-8/C-9, unequivocally confirmed the connectivity of Me-18 to C-6 and Me-19 to C-8, respectively. The construction of the six-membered ring A was also confirmed. Furthermore, the positional assignment of the associated olefinic carbon (C-3) was substantiated through attenuated COSY coupling between H-3 and H-4, supported by HMBC signals from H-3 to C-4/C-5/C-9. In a similar manner, parallel analysis of HMBC correlations from H-1 to C-2/C-3/C-9/C-10 supported the second six-membered ring B, with branching (Me-16) at C-1 confirmed through COSY (H_3_-16 and H-1) and HMBC (H_3_-16 → C-1/C-2/C-10) interactions. Concurrently, the placement of two singlet methyl groups (Me-17, Me-15) at C-2 and C-10 was deduced from HMBC cross-peaks from H_3_-17 to C-1/C-2/C-3, and from H_3_-15 to C-1/C-9/C-10, respectively. The methyl vinyl ketone moiety was rigorously characterized by COSY coupling between H-11 (*δ*_H_ 7.15) and H-12 (*δ*_H_ 5.97), complemented by HMBC correlations from H_3_-14 to C-12/C-13 and H-11 to C-9/C-10/C-13/C-15, alongside the diagnostic carbonyl resonance at *δ*_C_ 198.0 (C-13). Therefore, the planar structure of compound **1** was further assigned as a norditerpenoid with a 6/6-bicyclic fused ring system featuring an α,β-unsaturated methyl vinyl ketone sidechain at C-10.

**Figure 3:**
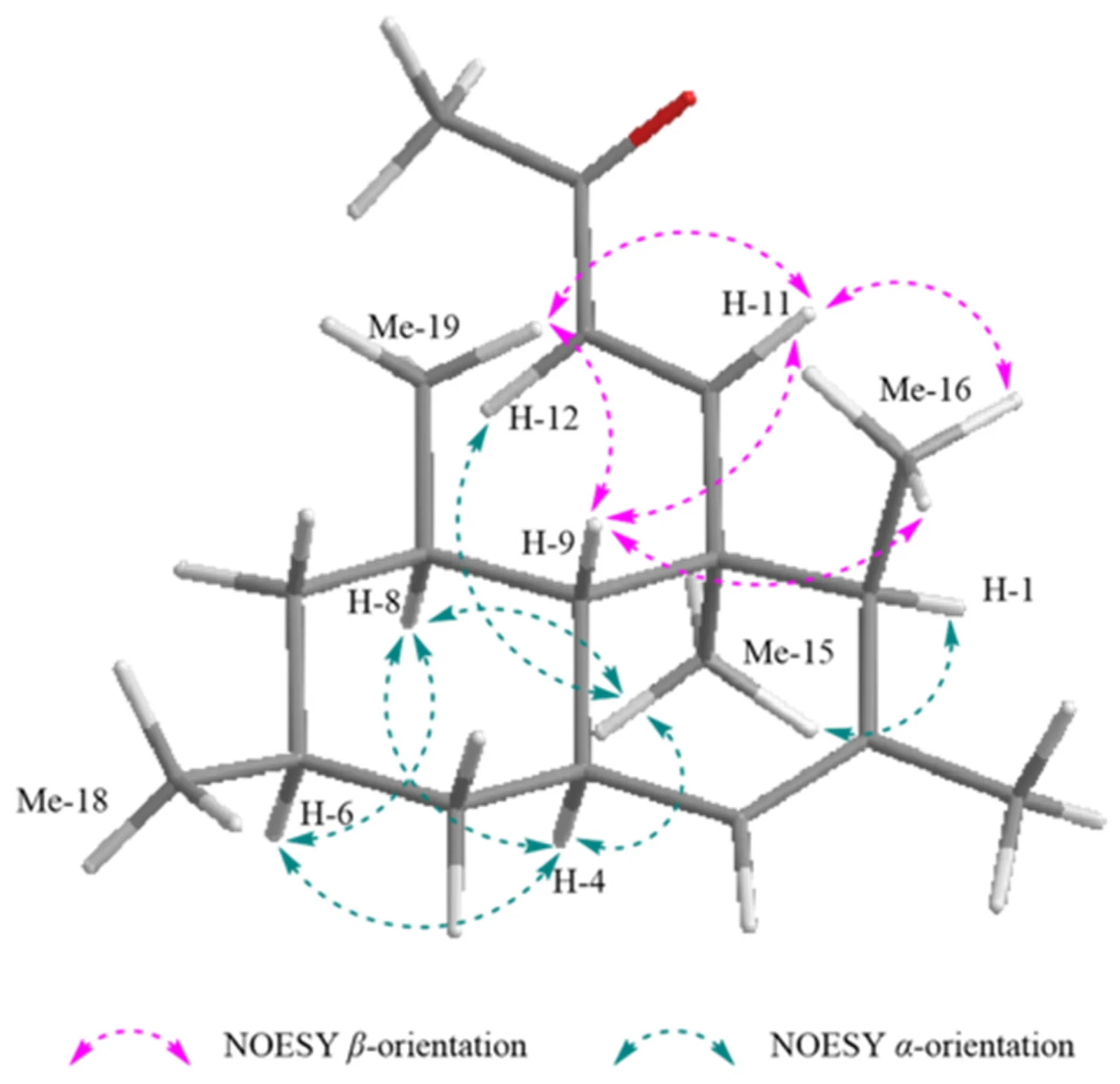
Key NOESY correlations of **1**.

**Figure 4:**
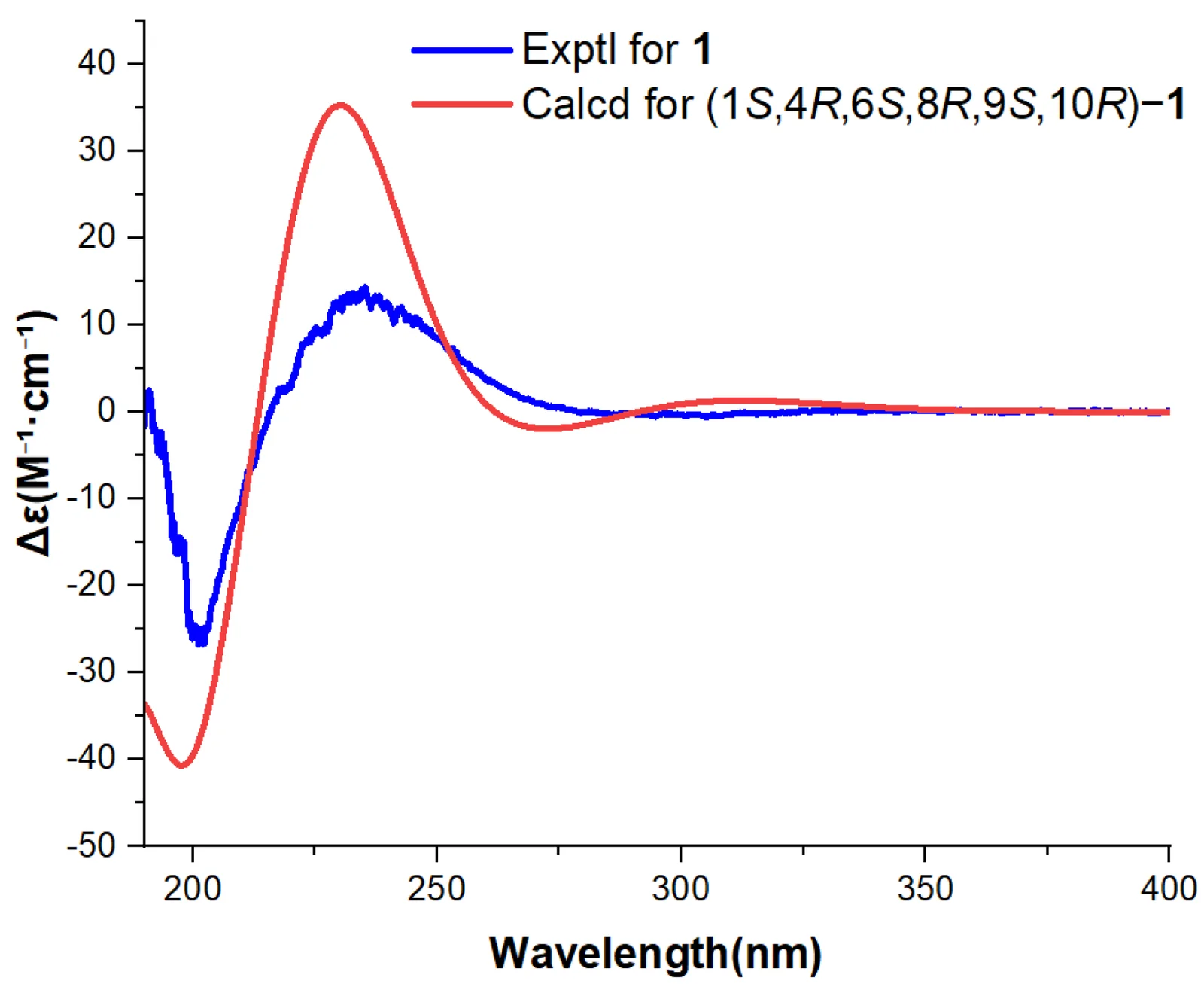
Comparisons between CD spectra measured in MeOH and calculated at the PBE1PBE/def2-TZVP/PCM level of theory for compound **1**.

To ascertain the relative configuration of compound **1**, we performed a comprehensive analysis combining *J*-based coupling constants with NOESY experiments. The *E*-configuration of the C11–C12 double bond (*J*_11, 12_ = 16.6 Hz) was confirmed by characteristic transoid coupling. Critical spatial relationships emerged from NOESY interactions (Figure 3): pronounced correlations between H-11 and H-9/H_3_-16/H_3_-19 localized these protons to the β-face, while weaker but diagnostic H-9 ↔ H_3_-16/H_3_-19 NOEs reinforced their axial β-orientation. Contrastingly, NOE networks linking H-4/H-6/H-8 on the α-face, complemented by H_3_-15 ↔ H-1/H-12 interactions, defined the opposing spatial topology. Finally, combined with ECD computational analysis (Figure 4), the absolute configuration of compound **1** was unambiguously established as 1*S*,4*R*,6*S*,8*R*,9*S*,10*R*. The calculated ECD curve of this enantiomer closely matched the experimental data, confirming the absolute configuration of nordichloridene (**1**).

Compound **2**, 9-chloro-1,2,3,4-tetrahydroacridine, was isolated as a pale-yellow powder through natural product extraction, marking the first reported occurrence of this acridine derivative in nature, supported by ^1^H/^13^C NMR data (Table S3). These spectral features align with reported tricyclic acridine derivatives [15], confirming the compound’s structural identity.

### 2.2. Antiepileptic Activity

**Figure 5:**
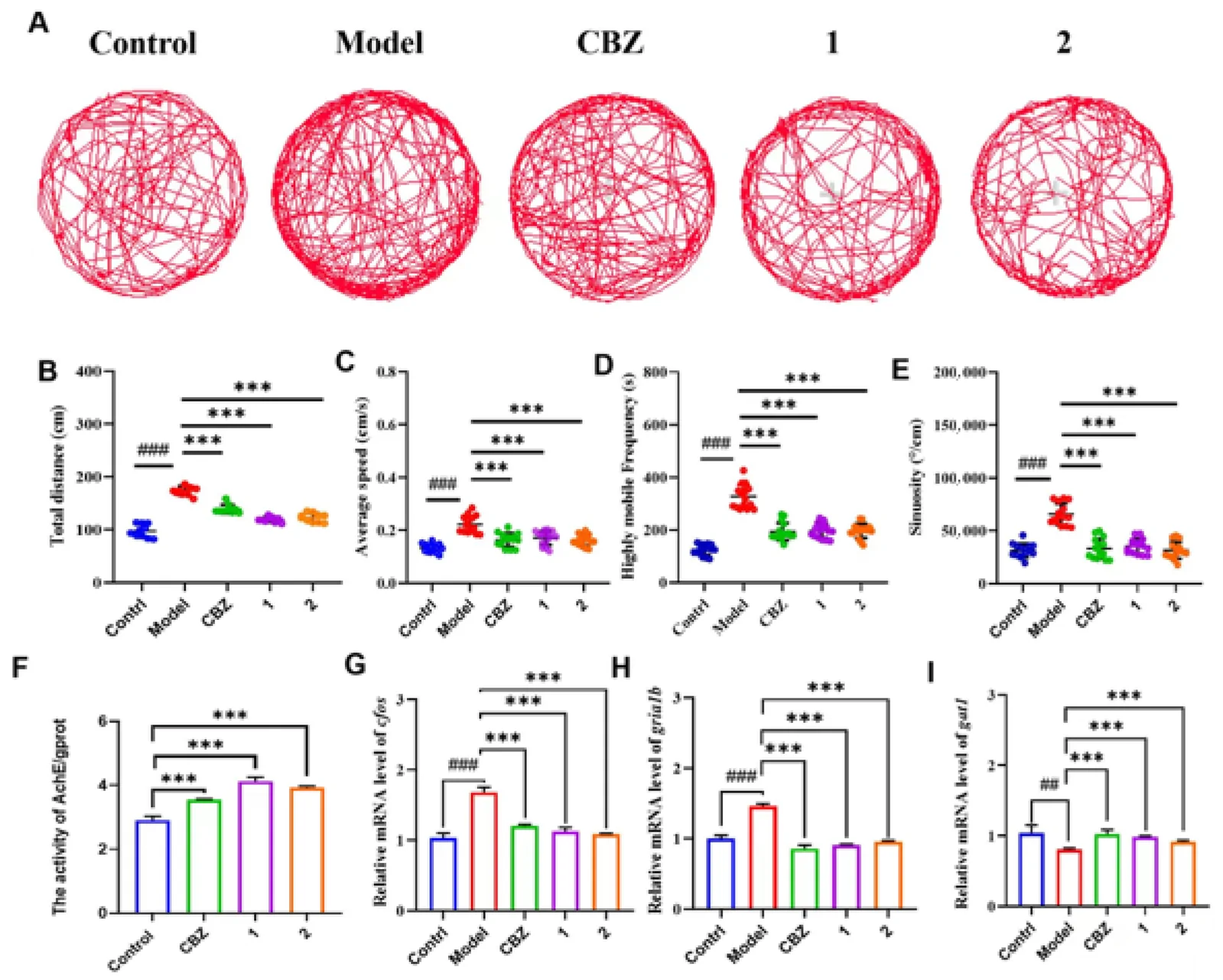
Antiepileptic activities of compounds **1** and **2** were assessed in the zebrafish model. (**A**) Representative movement tracks of zebrafish at 6 dpf. (**B**–**E**) Quantification of behavioral parameters including total distance traveled (**B**), average speed (**C**), highly mobile frequency (**D**), and sinuosity (**E**) in the different experimental groups (Control, Model, CBZ, compound **1**, and compound **2**). (**F**) Measurement of AChE activity in zebrafish larvae across groups. (**G**–**I**) Relative mRNA expression levels of epilepsy-related genes (*c-fos*, *gria1b*, and *gat1*) in zebrafish larvae. *p* ^##^ < 0.01 (significant) and *p* ^###^ < 0.001 (significant) compared with the control group; *p* *** < 0.001 (significant) compared with the model group.

In this study, the antiepileptic potential of compounds **1** and **2** was evaluated using a PTZ-induced zebrafish seizure model (Figure 5). Multiple parameters were assessed, including locomotor activity, AChE levels, and the expression of key epilepsy-related genes. PTZ administration induced pronounced hyperactivity in zebrafish, manifested by marked increases in total distance traveled, average speed, high mobility frequency, and movement sinuosity compared to the control group. Treatment with compounds **1** and **2** at 5.0 mg/L effectively attenuated these behavioral abnormalities, restoring locomotor parameters to levels comparable to or exceeding those observed in the 50 μM CBZ-treated group, indicating substantial suppression of seizure-associated hyperactivity (Figure 5A–E). Analysis of AChE activity revealed distinct differences across treatment groups (Figure 5F). The CBZ-treated group exhibited notably elevated AChE activity compared to the control group (*p* < 0.001). Notably, treatment with compounds **1** and **2** at 5.0 mg/L induced further increases in AChE activity (*p* < 0.001), with compound **1** exhibiting the most pronounced effect. These findings suggest that the antiepileptic effects of these compounds might be mediated through cholinergic pathway modulation, which plays a crucial role in regulating neuronal excitability and seizure susceptibility. This observation highlighted compounds **1** and **2**, particularly compound **1**, as promising therapeutic candidates targeting AChE regulation.

Gene expression analysis revealed that PTZ-induced seizures markedly upregulated *c-fos* and *gria1b* (an AMPA-type glutamate receptor subunit), both key modulators of neuronal excitability and glutamatergic signaling, while *gat1* (glutamine amidotransferase 1), crucial for GABAergic inhibition, was substantially downregulated in the PTZ model (Figure 5G–I). Treatment with compounds **1** and **2** at 5.0 mg/L effectively normalized these alterations, restoring *c-fos*, *gria1b*, and *gat1* expression to levels comparable to the control group. These results indicate that the compounds helped restore the balance between excitatory glutamatergic and inhibitory GABAergic neurotransmission, essential for maintaining normal neuronal excitability and preventing seizures.

Notably, compound **2** has been identified as an important precursor in the development of various compounds with potential for treating neurodegenerative diseases, such as Alzheimer’s disease [16], suggesting its broader neuroprotective properties. However, to the best of our knowledge, compound **2** has not been previously evaluated for its antiepileptic activity, making its current evaluation an important step toward expanding its therapeutic potential. In contrast, while compound **1** shares structural similarities with known compounds that have been explored in diverse pharmacological contexts, no studies have yet demonstrated its potential as an antiepileptic lead compound. The findings of this study therefore represent a novel investigation into the antiepileptic activity of compound **1** in epilepsy models, offering new insights into its therapeutic applicability. These results highlight the potential of both compounds as antiepileptic candidates, warranting further investigation to optimize their pharmacological profiles for clinical use.

## 3. Materials and Methods

### 3.1. General Experimental Procedures

MPLC was run on a FLEXA purification system fitted with an ODS column (Santai Technologies, Inc., Changzhou, China). Preparative HPLC was carried out on a Waters 600 system (Milford, MA, USA) coupled with a Waters 2996 photodiode array detector and a YMC-Pack ODS-A preparative reverse-phase C18 column (250 × 10 mm, 5 μm, Tokyo, Japan). CD spectra were measured on a JASCO J810 spectrometer (JASCO, Inc., Tokyo, Japan). Optical rotation values were determined with a PerkinElmer polarimeter (PerkinElmer, Waltham, MA, USA). NMR experiments were carried out on a Bruker AVANCE 600 MHz spectrometer (Bruker Biospin AG, Fällanden, Switzerland), and TMS was used as the internal reference. HRESIMS analysis was performed on a Waters Xevo G2-XS QTof mass spectrometer (Milford, MA, USA). Column chromatography was accomplished using silica gel (200–300 mesh, Qingdao Marine Chemical Factory, Qingdao, China). Locomotor trajectories were tracked with a DanioVision observation chamber (Noldus Information Technology, Wageningen, The Netherlands). PCR was run on an instrument supplied by Hangzhou Langji Scientific Instrument Co., Ltd. (Hangzhou, China). PTZ (M19454), CBZ (D129539), and DMSO (A610163) were sourced from Shanghai Meryer Chemical Technology Co., Ltd. (Shanghai, China), Shanghai Aladdin Biochemical Technology Co., Ltd. (Shanghai, China), and Shanghai Sangon Biotechnology Co., Ltd. (Shanghai, China), respectively.

### 3.2. Fungal Material

The fungal strain NBU3282 was isolated from *Caulerpa algae* collected at a depth of 10 m off the coast of Ningbo, China. The algal samples were collected by trawling in compliance with local regulations, with no specific permits required. A voucher specimen has been deposited on potato dextrose agar (PDA) at Ningbo University, China. Taxonomic identification was achieved through morphological examination combined with PCR amplification and sequencing of the ITS regions. The strain NBU3282 was identified as *Chloridium* sp. based on 99.8% sequence identity with the reference sequence (NCBI Accession No. OQ507332).

### 3.3. Fermentation, Extraction and Isolation

The fungal isolate NBU3282 was first grown on PDA agar plates at 28 °C for 3 days. The resulting mycelium was subsequently disrupted and used to inoculate ten 1 L Erlenmeyer flasks, each holding 300 mL of sterile potato dextrose broth (PDB; composed of 200 g potato pieces, 20 g glucose, 35 g sea salt, and 1000 mL distilled water). These flasks were maintained on a rotary shaker (28 °C, 150 rpm) for 4 days to prepare seed cultures. For scale-up fermentation, 400 Erlenmeyer flasks were filled with a solid rice substrate (90 g rice, 3 g sea salt, and 110 mL water per flask) and sterilized by autoclaving at 121 °C for 15 min. Each flask then received 5–7 mL of seed culture and was kept under static conditions at 28 °C for 30 days. All manipulations were performed aseptically. The fermented rice substrate was exhaustively extracted three times with EtOAc, and the combined extracts were concentrated under reduced pressure to give a crude residue (23.97 g). The extract was initially fractionated by silica gel MPLC (200–300 mesh) with a petroleum ether–EtOAc gradient (20:1 to 0:20, *v*/*v*), affording eight fractions (Fr. A–H).Fr. D was then applied to an ODS MPLC column and eluted with MeOH–H_2_O (20:80 to 100:0, *v*/*v*) at 15 mL/min for 2 h, giving ten subfractions (Fr. D.1–D.10). Subfraction D.4 was further purified by semi-preparative HPLC (YMC-Pack ODS-A, 250 × 10 mm, 5 μm) using MeCN–H2O (50:50, *v*/*v*) at 2 mL/min, furnishing compounds 1 (3.2 mg) and 2 (1.5 mg).

Nordichloridene (**1**): white amorphous powder; [*α*_D_] ^25^ = +72.2 (*c* 0.025, MeOH); UV (MeOH) *λ*_max_ (log *ε*) = 195.6 (0.53), 234.7 (0.43) nm; IR (KBr) *v*_max_ 2854.9, 1715.0, 1464.8, 1355.2 cm^−1^; ^1^H NMR (DMSO-*d*_6_, 600 MHz) and ^13^C NMR (DMSO-*d*_6_, 150 MHz) data, see Table 1; HRESIMS: *m*/*z* 275.2376 [M + H]^+^ (calculated for C_19_H_31_O, 275.2375).

9-Chloro-1,2,3,4-tetrahydroacridine (**2**): pale yellow powder; [*α*_D_] ^25^ = 0 (*c* 0.025, MeOH); ^1^H NMR (DMSO-*d*_6_, 600 MHz) and ^13^C NMR (DMSO-*d*_6_, 150 MHz) data, see Table S3.

### 3.4. Computational Details

Conformational analysis of compound **1** was performed via the mixed torsional/low-mode sampling method in MacroModel (version 12.8) with the MMFF molecular mechanics force field under vacuum conditions. Conformers within an energy range of 5.02 kcal/mol from the lowest-energy conformer were retained. Geometry optimization and frequency calculations were carried out in the gas phase at the B3LYP/6-31G(d) level using Gaussian 16 [17]. All optimized structures were confirmed to have no imaginary frequencies (NIMAG = 0). The corresponding Boltzmann distributions were evaluated by means of the Shermo (version 2.3.4) [18] software. Only conformers with a Boltzmann distribution probability exceeding 1% were selected for subsequent ECD calculations., ECD calculations were implemented in methanol (MeOH) at the PBE1PBE/def2-TZVP level using the polarizable continuum model (PCM). The theoretical ECD spectrum was generated by Boltzmann-weighted averaging of the calculated spectra using Multiwfn [19]. The resulting spectrum was then simulated with SpecDis (version 1.70) [20] using a Gaussian half-bandwidth of 0.32 eV, UV-shifted by −10 nm to match the experimental data, and plotted with Origin 2022.

### 3.5. Zebrafish Behavioral Tracking and Analysis

Adult wild-type AB strain zebrafish (*Danio rerio*) were sourced from the China Zebrafish Resource Center (Wuhan, Hubei, China) and maintained under standard laboratory conditions (14:10 h light:dark cycle, 28 ± 0.5 °C). Fish were fed *Artemia salina* (brine shrimp) twice daily. Ethical approval was granted by the Laboratory Animal Welfare and Ethics Committee of Gannan Normal University (approval code: gnnu2022-0628).

Zebrafish larvae at 5 days post-fertilization (dpf) were randomly assigned to experimental groups (*n* = 100 per group). The sample size was determined through power analysis to ensure sufficient statistical reliability. Experimental groups included a control group, a PTZ-treated group, compound-treated groups (5.0 mg/L), and a positive control group treated with carbamazepine (CBZ, 50 μM). The concentration of dimethyl sulfoxide (DMSO) was maintained below 0.1%, with equal volumes added to all groups to minimize solvent-induced effects on zebrafish behavior. All groups, except for the control group, were exposed to 10 mM PTZ for 30 min [21]. Behavioral recordings were conducted in 96-well plates under dark conditions (0 lux) to minimize external stimuli. After PTZ exposure, larvae were acclimatized in darkness for 10 min before 15-min behavioral recording. Behavioral data were analyzed using EthoVision XT 10.0 software with standardized settings (sampling rate: 30 Hz, duration: 15 min). Key parameters included total distance traveled, average speed, highly mobile frequency (movements exceeding 15 mm/s per minute), and sinuosity (path length/Euclidean distance)—all indicative of seizure-like behaviors.

Mortality was monitored daily throughout the experiment. If mortality exceeded 10% in any group, compound concentration or PTZ treatment conditions were adjusted. The optimal treatment concentration (5.0 mg/L) was determined through preliminary experiments, and CBZ (50 μM) served as a positive control to validate the PTZ-induced seizure model and assess the compounds′ antiepileptic activity [22].

### 3.6. Acetylcholinesterase (AChE) Activity Assessment

AChE activity was measured using a commercial assay kit (Sigma-Aldrich, St. Louis, MO, USA, Catalog Number A3272) based on the Ellman method, with thiocholine production monitored at 405 nm. Total protein was extracted from pools of 60 zebrafish embryos per treatment group using ice-cold RIPA buffer (150 mM NaCl, 1% NP-40, 0.5% sodium deoxycholate, and 0.1% SDS in 50 mM Tris-HCl, pH 7.4). Samples were homogenized on ice with a motor-driven glass-Teflon homogenizer (Wheaton, 15 mL) at 1000 rpm for 2 min, followed by centrifugation (12,000 rpm, 10 min, 4 °C) to obtain the soluble protein fraction from the supernatant. During the entire extraction process, samples were maintained at 4 °C to prevent protein degradation. Protein concentrations were quantified using the Bradford assay with Coomassie Brilliant Blue G-250 against a bovine serum albumin (BSA, ≥98% purity, Sigma-Aldrich) standard curve (0–1 mg/mL). Absorbance was measured at 595 nm on a PerkinElmer multimode microplate reader (Waltham, MA, USA). Each sample was assayed in triplicate. Three independent biological replicates were used, and the data are reported as mean ± standard error of the mean (*SEM*). Enzyme activity was normalized against total protein and expressed in U/mg protein. For multiple comparisons, one-way ANOVA with Tukey’s post hoc test was employed. Normality was evaluated with the Shapiro−Wilk test. The significance threshold was *p* < 0.05.

### 3.7. Analysis of Epilepsy-Related Gene Expression

For each treatment group, total RNA was prepared from pooled samples of 30 zebrafish larvae. Following collection, samples were snap-frozen in liquid nitrogen and ground with a sterile, RNase-free mortar and pestle under RNase-free conditions. RNA was then purified with a spin column-based kit (B518651, Sangong Biotech Co., Ltd., Shanghai, China) following the supplier’s instructions, incorporating an additional on-column DNase I digestion (15 min, room temperature) to remove residual genomic DNA. RNA yield and purity were determined on a NanoDrop One UV−Vis spectrophotometer (Thermo Fisher Scientific, Waltham, MA, USA), applying thresholds of 1.8–2.0 for the A260/A280 ratio and >1.8 for A260/A230. Integrity was confirmed by 1% agarose gel electrophoresis (100 V, 30 min), which displayed distinct 28S and 18S rRNA bands at an approximate 2:1 ratio. To reduce freeze−thaw cycling, purified RNA was divided into aliquots and kept at −80 °C in RNase-free tubes supplemented with 1 µL RNase inhibitor per 50 µL of RNA solution. For cDNA synthesis, total RNA samples were normalized to 1.0 µg in a 20 µL reaction volume using the *MightyScript Plus First Strand cDNA Synthesis Master Mix* (B639252, Sangong Biotech Co., Ltd.). The reaction conditions included initial RNA/primer denaturation at 65 °C for 5 min followed by immediate cooling on ice, reverse transcription at 42 °C for 60 min, and enzyme inactivation at 85 °C for 5 min. The efficiency of reverse transcription was verified by amplifying the housekeeping gene *elongation factor 1α* (*ef1α)* using standard PCR, with parallel reactions excluding reverse transcriptase (-RT controls) to confirm the absence of genomic DNA contamination.

Quantitative real-time PCR was performed on a qTOWER 3G instrument (Analytik Jena, Jena, Germany) using *SGExcel FastSYBR Mixture* (B532955, Sangong Biotech Co., Ltd.). Each 20 µL reaction contained 2 µL of diluted cDNA (1:10), 0.5 µM of each primer, 10 µL of SYBR Green Master Mix, and nuclease-free water. All reactions included no-template controls (NTC) and were performed in technical triplicates, with inter-plate calibrators used for multi-plate experiments. The amplification protocol consisted of initial denaturation at 94 °C for 180 s, followed by 40 cycles of denaturation (94 °C, 5 s) and combined annealing/extension (60 °C, 30 s). Melting curve analysis was performed immediately after amplification by heating from 60 °C to 95 °C (0.5 °C increments, 5 s per step) with continuous fluorescence monitoring. Product specificity was confirmed by both melting curve analysis showing single peaks and 2% agarose gel electrophoresis displaying single bands of expected sizes.

Primer pairs (sequences provided in Table S4) were designed using *Primer-BLAST* with the following criteria: amplicon size 80–150 bp, primer length 18–22 nucleotides, GC content 45–55%, and melting temperature 58–62 °C. Primer specificity was validated in silico against the zebrafish genome (*GRCz11*) and experimentally through melt curve analysis. Standard curves using serial dilutions of pooled cDNA (1:5, 1:25, 1:125, 1:625) confirmed primer amplification efficiencies between 90% and 110% (R^2^ > 0.99) for all primer pairs. The stability of *ef1α* expression under experimental conditions was verified using BestKeeper software (version 1.0) and RefFinder analysis. Relative gene expression was calculated using the 2^−ΔΔCt^ method and presented as the mean ± *SEM* from three independent biological replicates, each performed in technical triplicate.

## 4. Conclusions

In summary, this study successfully isolated nordichloridene (**1**), a previously undescribed bicyclic norditerpenoid, and 9-chloro-1,2,3,4-tetrahydroacridine (**2**), a compound reported solely through synthetic methods, from the marine algae-derived fungus *Chloridium* sp. NBU3282. Structurally, nordichloridene (**1**) is distinguished by its unique bicyclic norditerpenoid framework incorporating a rare methacrylic ketone moiety, rendering it exceptionally uncommon among fungal metabolites. Both compounds exhibited significant antiepileptic activity in a PTZ-induced zebrafish model, showing particularly promising effects in mitigating PTZ-induced hyperactivity, in moderately enhancing AChE activity, and in restoring the balance between excitatory and inhibitory neurotransmission. These findings highlight the potential of marine fungi as a valuable source of structurally diverse and bioactive compounds, offering new avenues for antiepileptic drug discovery. Future efforts in natural product analogue synthesis, synthetic medicinal chemistry, and biosynthetic exploration of these compounds are encouraged to facilitate further antiepileptic drug discovery and development.

## Figures and Tables

**Table 1:** H and ^13^C NMR data of compound **1** (600 MHz, 150 MHz, DMSO-*d*_6_).

Position	*δ*_C,_ Type	*δ*_H_ (*J* in Hz)	HMBC	COSY
C-1	49.2, CH	1.45, q (7.0)	C-2, 3, 9, 10, 16, 17	H_3_-16
C-2	135.3, C			
C-3	124.7, CH	5.07, br s	C-1, 4, 5, 9, 17	H-4
C-4	39.6, CH	1.80, m	C-2, 3, 5, 6, 8, 9	H-3, 9
C-5	42.1, CH_2_	0.76, overlapped	C-6, 7	H-4, 6
		1.72, m	C-4, 7, 9, 18	H-4, 6
C-6	32.5, CH	1.55, m	C-7	H-5a
C-7	46.9, CH_2_	0.86, overlapped	C-5	H-6, 8
		1.59, overlapped	C-5	
C-8	34.8, CH	1.41, m		H-7a, 9, H_3_-19
C-9	47.3, CH	1.22–1.30, overlapped	C-1, 4, 7, 8, 15	H-4, 8
C-10	41.3, C			
C-11	161.6, CH	7.15, d (16.6)	C-9, 10, 12, 13, 15	H-12
C-12	124.5, CH	5.97, d (16.6)	C-10, 11, 13, 14	H-11
C-13	198.0, C			
C-14	27.2 CH_3_	2.21, s	C-12, 13	
C-15	17.3, CH_3_	1.01, s	C-1, 10, 11	
C-16	16.2, CH_3_	0.94, d (7.0)	C-1, 2, 10	H-1
C-17	21.8, CH_3_	1.61, s	C-1, 2, 3	
C-18	22.3, CH_3_	0.85, d (6.5)	C-5, 6, 7	H-6
C-19	22.2, CH_3_	0.80, d (6.8)	C-7, 8, 9	H-8

## Data Availability

The original contributions presented in this study are included in the article. Further inquiries can be directed to the corresponding author.

## Supplementary Materials

The following supporting information can be downloaded at https://www.mdpi.com/article/10.3390/md24100369/s1, Figure S1: HRESIMS spectrum of compound **1**; Figure S2: UV spectrum of compound **1**; Figure S3: Infrared spectroscopy (IR) of compound **1**; Figure S4: ^1^H NMR spectrum (600 MHz, DMSO-*d*_6_) of compound **1**; Figure S5: ^13^C NMR spectrum (150 MHz, DMSO-*d*_6_) of compound **1**; Figure S6: DEPT-135 spectrum (150 MHz, DMSO-*d*_6_) of compound **1**; Figure S7: HSQC spectrum (DMSO-*d*_6_) of compound **1**; Figure S8: HMBC spectrum (DMSO-*d*_6_) of compound **1**; Figure S9: ^1^H-^1^H COSY(DMSO-*d*_6_) spectrum of compound **1**; Figure S10: NOESY(DMSO-*d*_6_) spectrum of compound **1**; Figure S11: The comparison between theoretical calculations and experimental measurements of ECD in MeOH; Figure S12: ^1^H NMR (600 MHz, DMSO-*d*_6_) spectrum of compound **2**; Figure S13: ^13^C NMR (150 MHz, DMSO-*d*_6_) spectrum of compound **2**; Table S1: Boltzmann weight >1% for diastereomer 1*S*,4*R*,6*S*,8*R*,9*S*,10*R* of compound **1**; Table S2: Cartesian coordinates of 1*S*,4*R*,6*S*,8*R*,9*S*,10*R*; Table S3: Comparison of ^1^H and ^13^C NMR data of compound **2** between the experimental values and literature values; Table S4: Primer sequences used in the study.
