# Supplementary material for: Discovery of a Novel Antiepileptic Bicyclic Norditerpenoid from the Marine Algae-Derived Fungus Chloridium sp. NBU3282
Source: Mar Drugs. 2026 Oct 21;24(10):369. doi: 10.3390/md24100369 (PMC13706009; doi:10.3390/md24100369)
Supplement: Supplementary file 1 [file marinedrugs-24-00369-s001.zip › marinedrugs-4603054-supplementary.pdf]

Discovery of a Novel Antiepileptic Bicyclic Norditerpenoid from the Marine Algae-Derived Fungus *Chloridium* sp. NBU3282

Pengcheng Zhou [1,2,] , Yuqi Su [1,2] , Yujia Li [2] , Wenhui Zhang [2] , Lixin Chen [3] , Yufeng Xie [3] , Wei Yuan [3] , Fangjian Feng [2] , Lijian Ding [1,2,4,*] and Zhengquan Wang [1*]

[1] *Department of Emergency Medicine, The Affiliated Yangming Hospital of Ningbo University (Yuyao People's Hospital), Ningbo University, Ningbo, Zhejiang, China*

[2] *School of Pharmacy, Health Science Center, Ningbo University, Ningbo 315211, China*

[3] *Jiangxi Provincial Key Laboratory of Synthetic Pharmaceutical Chemistry, School of Geography and Environmental Engineering, Gannan Normal University, Ganzhou, 341000, Jiangxi, China*

[4] *School of Pharmacy, Changsha Medical University, Changsha 410219, China.*

[*] Corresponding author. E-mail address: dinglijian@nbu.edu.cn (Lijian Ding) ;13777106152@163.com (Zhengquan Wang) .

Supplementary materials

Contents

Figure S1 HRESIMS spectrum of compound **1**. 3
Figure S2 UV spectrum of compound **1**. 3
Figure S3 Infrared spectroscopy (IR) of compound **1**. 4
Figure S4 ^{1}H NMR spectrum (600 MHz, DMSO-d_6) of compound **1**. 4
Figure S5 ^{13}C NMR spectrum (150 MHz, DMSO-d_6) of compound **1**. 5
Figure S6 DEPT-135 spectrum (150 MHz, DMSO-d_6) of compound **1**. 5
Figure S7 HSQC spectrum (DMSO-d_6) of compound **1**. 6
Figure S8 HMBC spectrum (DMSO-d_6) of compound **1**. 6
Figure S9 ^{1}H-^{1}H COSY (DMSO-d_6) spectrum of compound **1**. 7
Figure S10 NOESY (DMSO-d_6) spectrum of compound **1**. 7
Figure S11 The comparison between theoretical calculations and experimental measurements of ECD in MeOH. 8
Figure S12 ^{1}H NMR (600 MHz, DMSO-d_6) spectrum of compound **2**. 8
Figure S13 ^{13}C NMR (150 MHz, DMSO-d_6) spectrum of compound **2**. 9
Table.S1 Boltzmann weight >1% for diastereomer 1S,4R,6S,8R,9S,10R of compound **1**. 9
Table.S2 Cartesian coordinates of 1S,4R,6S,8R,9S,10R. 9
Table.S3 Comparison of ^{1}H and ^{13}C NMR data of compound **2** between the experimental values and literature values. 12
Table.S4 Primer sequences used in the study. 12

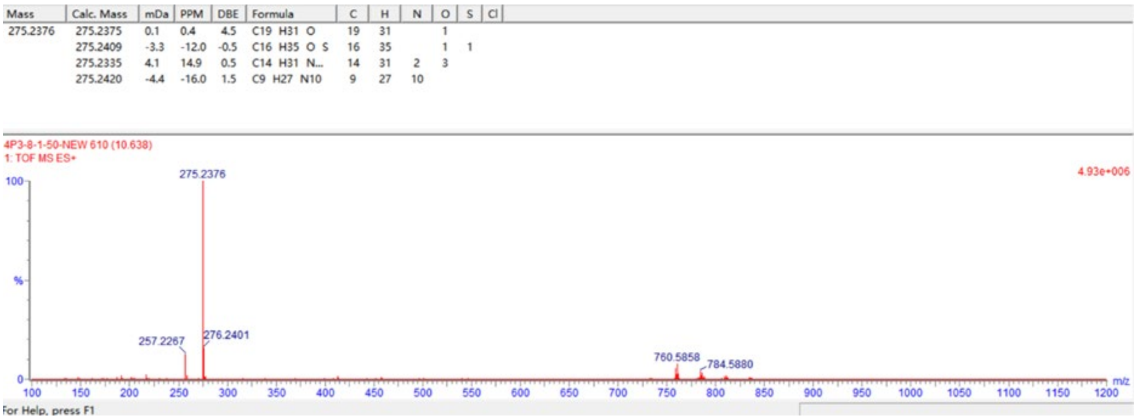

Figure S1 HRESIMS spectrum of compound **1**.

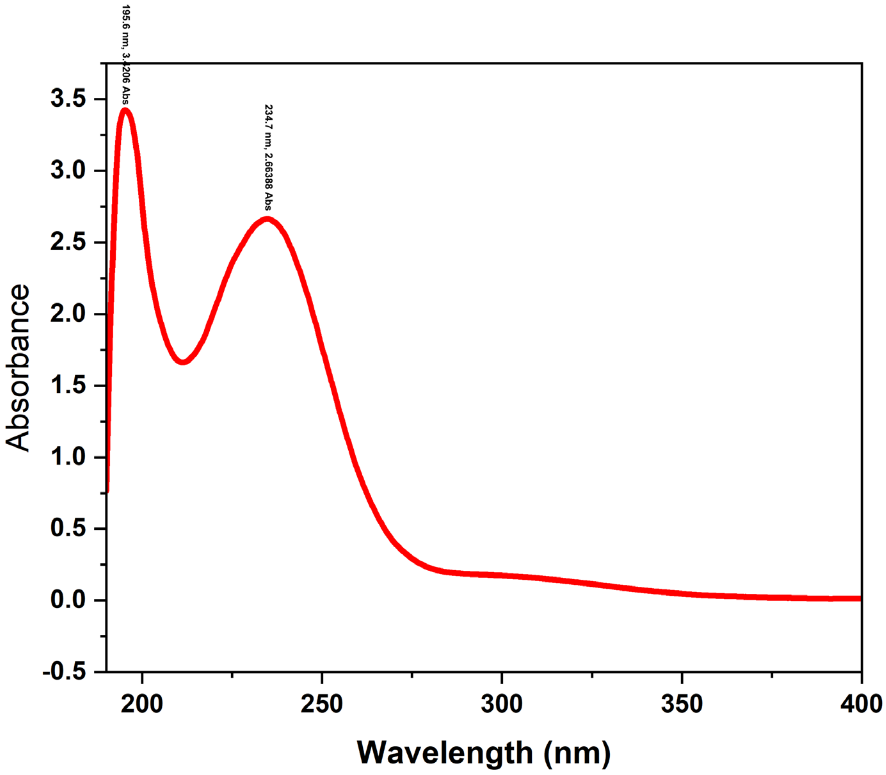

Figure S2 UV spectrum of compound **1**.

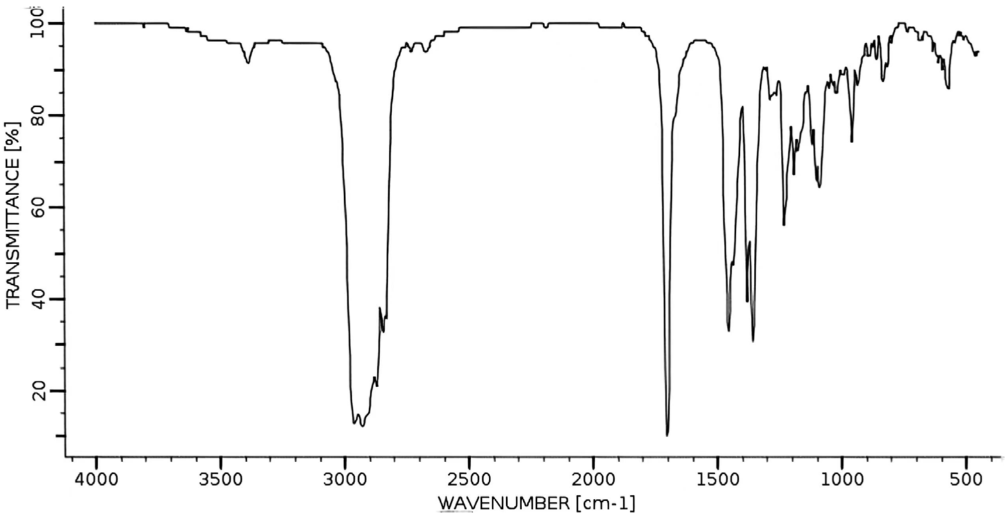

Figure S3 Infrared spectroscopy (IR) of compound **1**.

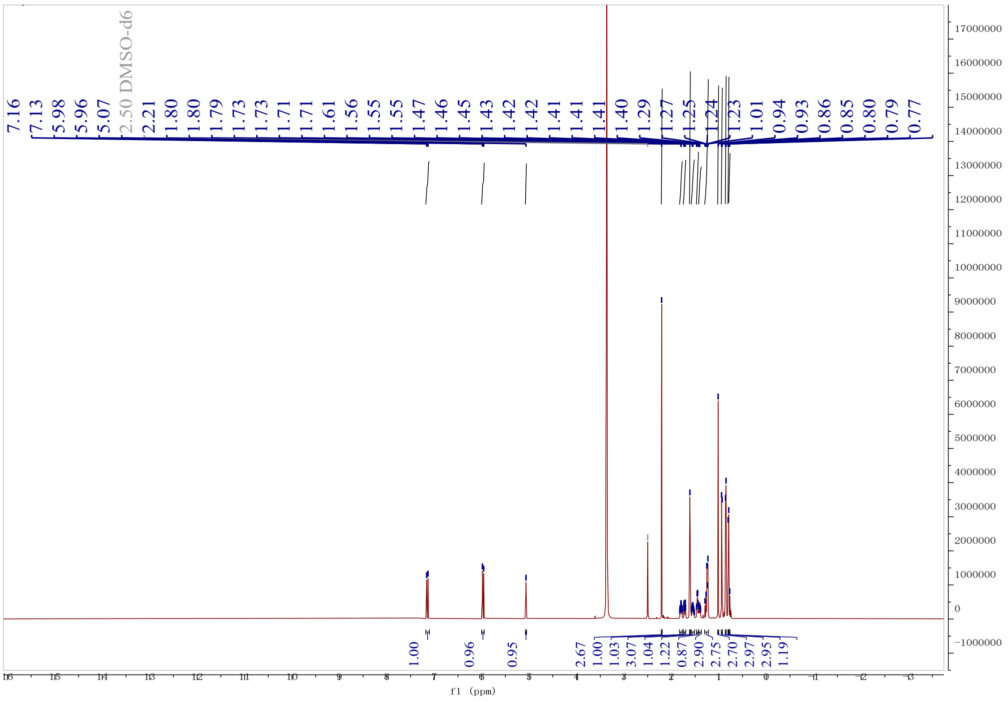

Figure S4 ^{1}H NMR spectrum (600 MHz, DMSO-d_6) of compound **1**.

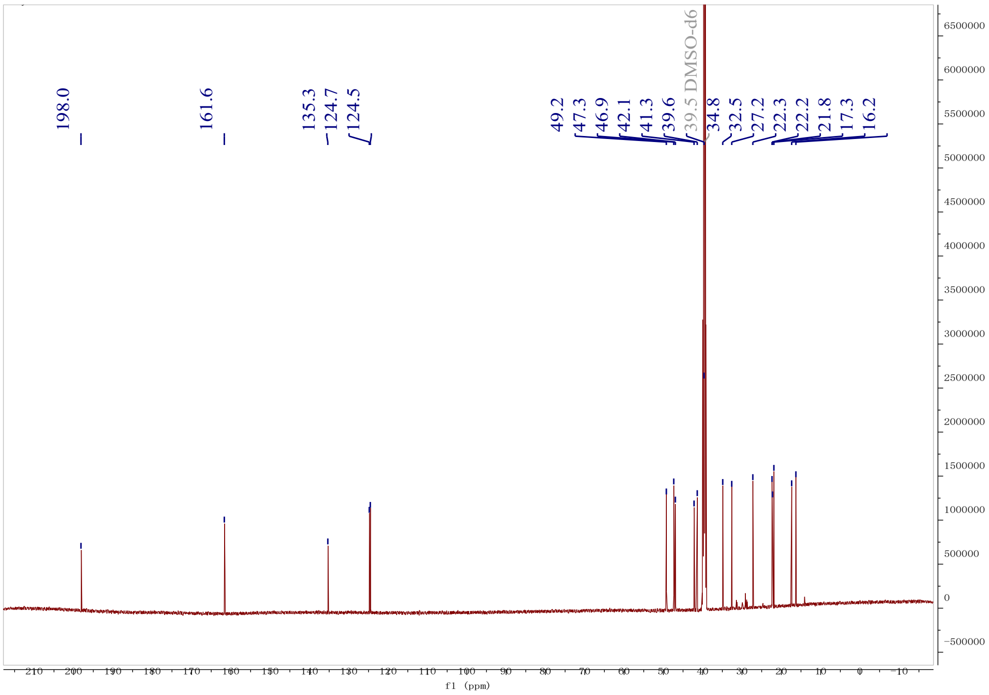

Figure S5 ^{13}C NMR spectrum (150 MHz, DMSO-d_6) of compound **1**.

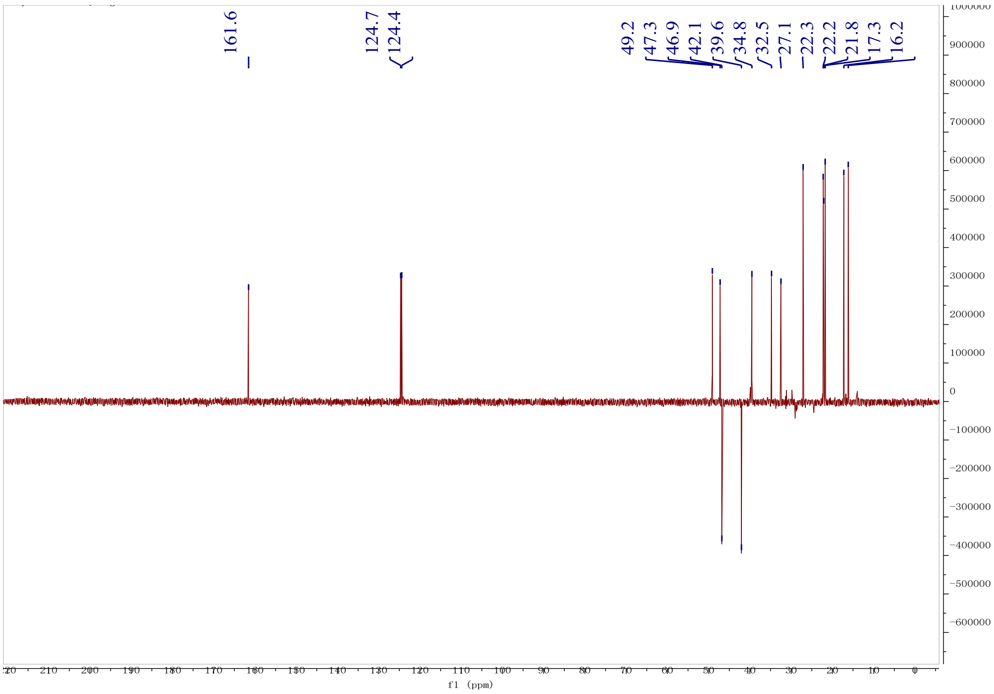

Figure S6 DEPT-135 spectrum (150 MHz, DMSO-d_6) of compound **1**.

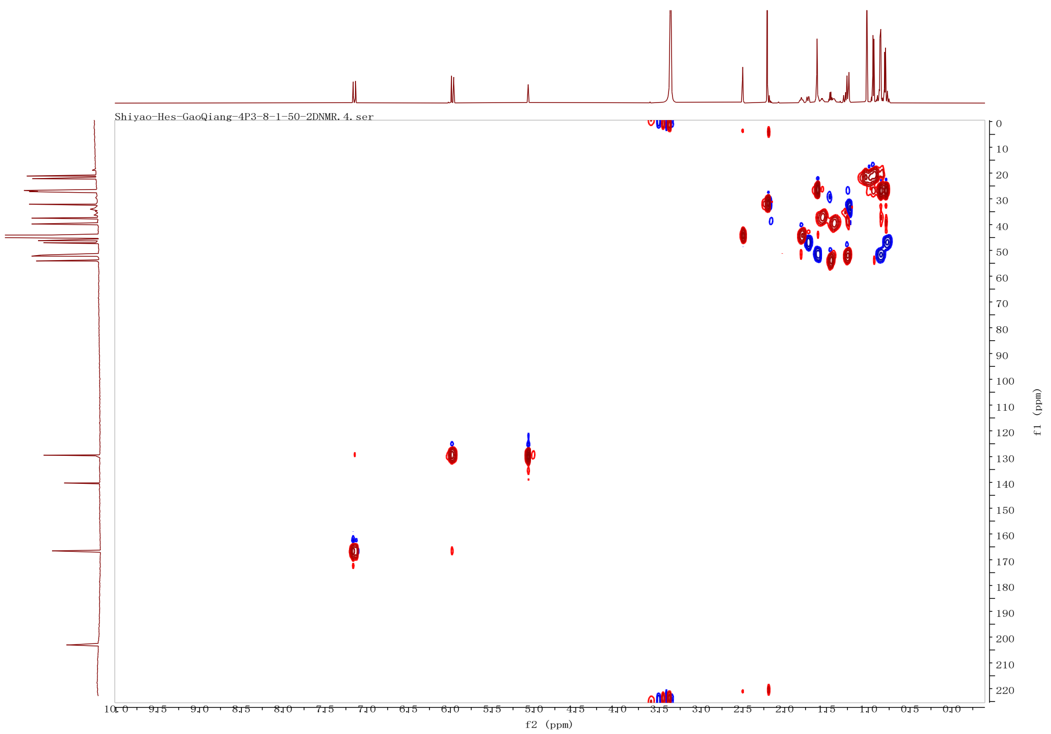

Figure S7 HSQC spectrum (DMSO-d_6) of compound **1**.

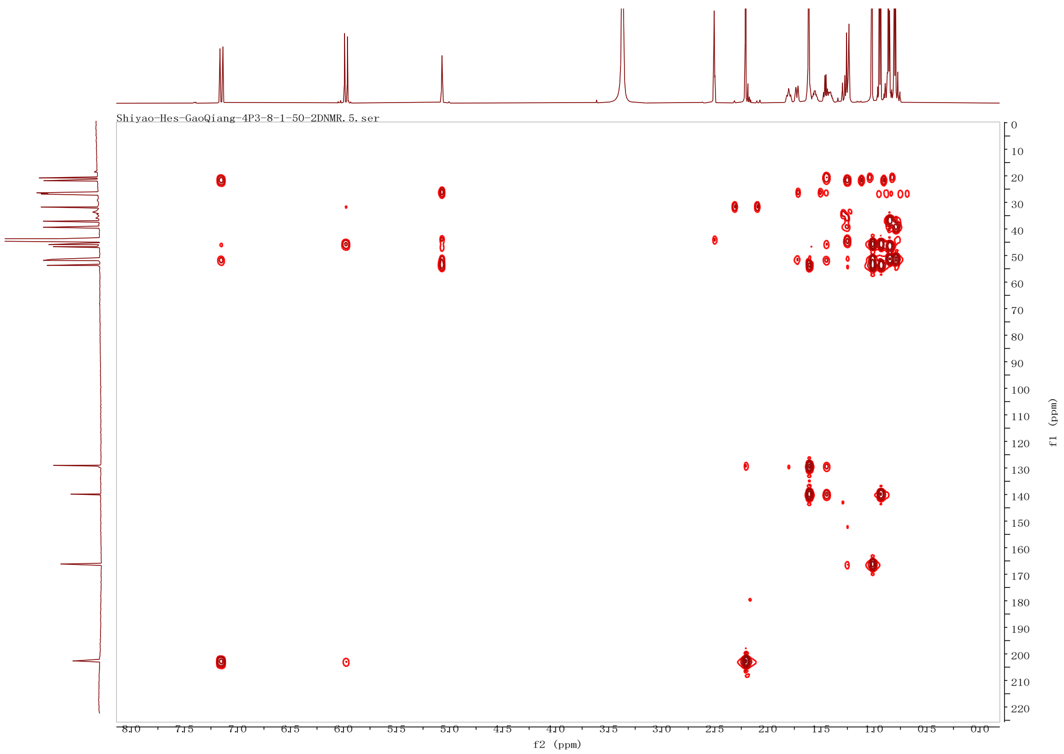

Figure S8 HMBC spectrum (DMSO-d_6) of compound **1**.

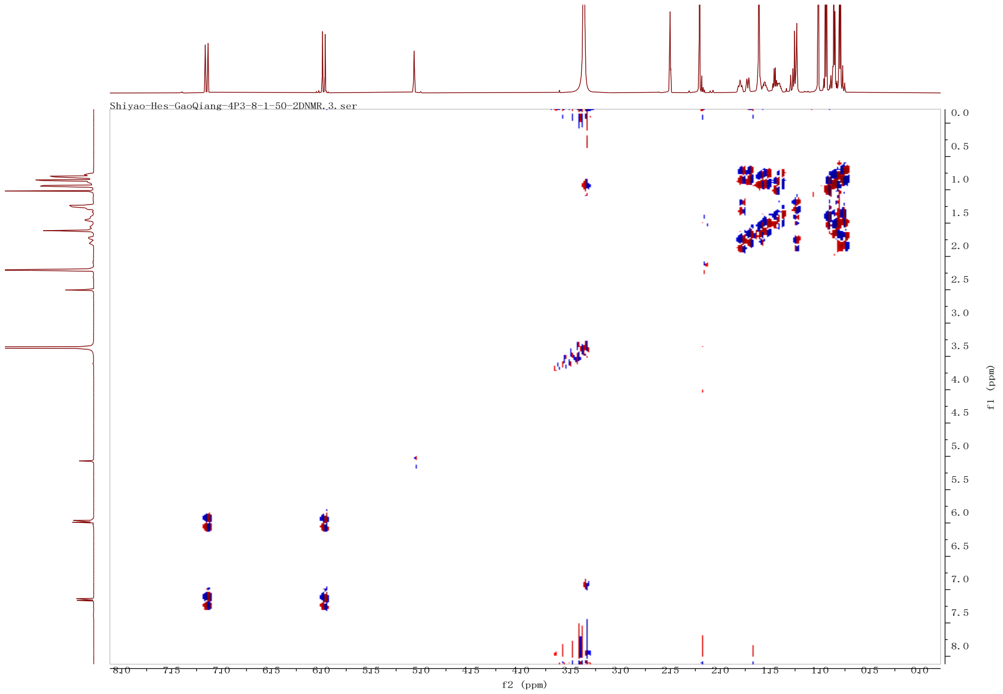

Figure S9 ^{1}H-^{1}H COSY(DMSO-d_6) spectrum of compound **1**.

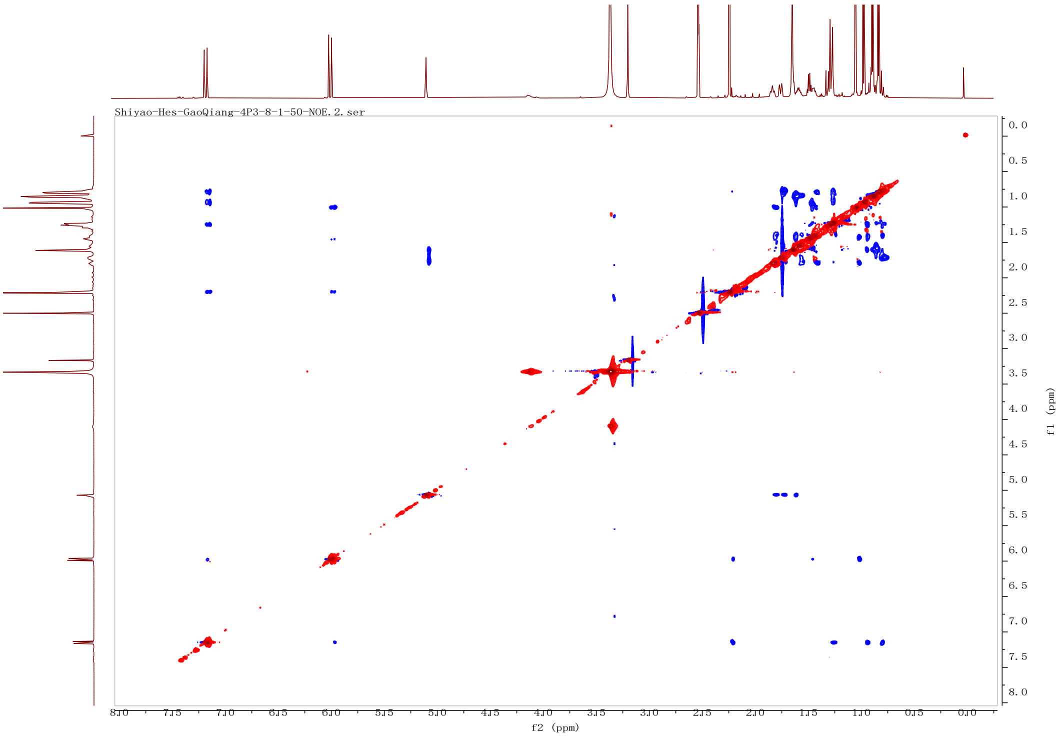

Figure S10 NOESY(DMSO-d_6) spectrum of compound **1**.

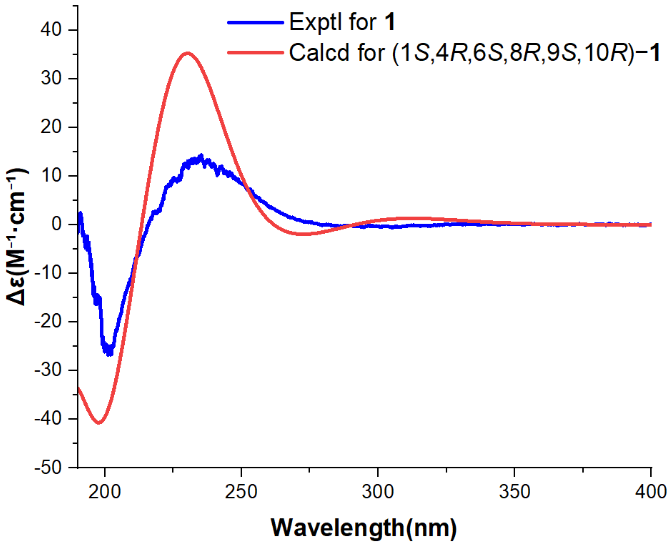

Figure S11 The comparison between theoretical calculations and experimental measurements of ECD in MeOH.

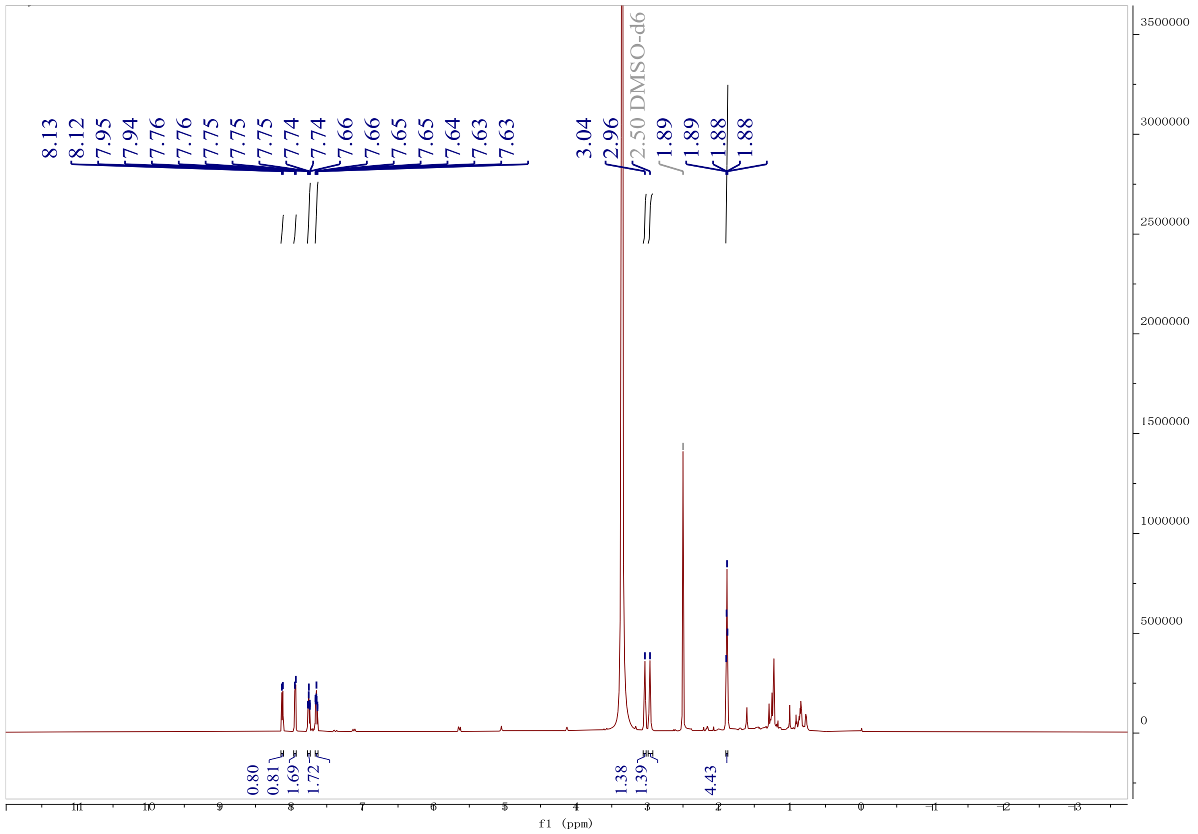

Figure S12 ^{1}H NMR (600 MHz, DMSO-d_6) spectrum of compound **2**.

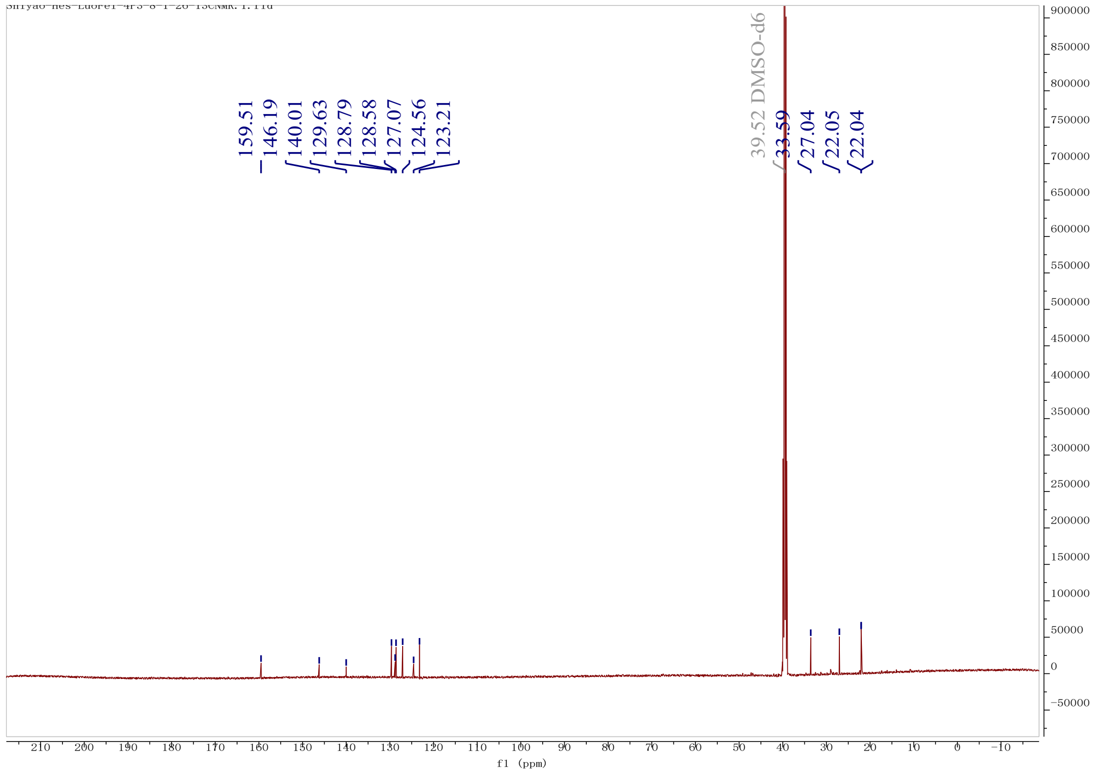

Figure S13 ^{13}C NMR (150 MHz, DMSO-d_6) spectrum of compound **2**.

Compound **1**

Table S1 Boltzmann weight >1% for diastereomer 1*S*,4*R*,6*S*,8*R*,9*S*,10*R* of compound **1**.

Conformers	Relative G (KJ/mol)	Boltzmann weight (%)
1	3.428	19.996
2	0.000	79.694

Table S2 Cartesian coordinates of 1*S*,4*R*,6*S*,8*R*,9*S*,10*R*

Conformer 1

0 1			
C	1.87215900	2.06119800	-0.23947300
C	0.73081400	2.70560100	0.01607900
C	2.00052900	0.57825300	-0.42780200
C	3.24165600	0.01252400	0.28027300
C	3.47762500	-1.45035700	-0.10768200
C	4.67331700	-2.05314400	0.63330500
C	0.93121300	-1.68112600	-0.52703600
C	-0.23147600	-2.64943100	-0.27740000

C	0.73756700	−0.22187900	−0.02874900
H	0.71362600	−0.26858800	1.07090800
O	−5.24648600	−0.89501500	−0.57111900
C	−4.18310700	−0.89790500	0.03434400
C	−2.99576600	−0.23736300	−0.55210900
C	−0.55949100	0.53657800	−0.46274000
C	−0.56725500	1.95429900	0.22933900
C	0.66571100	4.20755300	0.11316400
C	2.20155500	−2.26758200	0.12461600
C	−1.79479400	−0.17055700	0.04210300
C	−0.60644600	0.72861900	−1.98758700
H	2.16648600	0.40927200	−1.50636000
C	−4.05916900	−1.58160900	1.38866000
C	−0.90807000	1.92619400	1.73517500
H	2.78863000	2.63639400	−0.37684300
H	3.10946000	0.09528600	1.36920100
H	4.12339700	0.61569400	0.02529600
H	3.69597300	−1.47194800	−1.18701400
H	4.50051600	−2.05200700	1.71704200
H	4.85152100	−3.09015500	0.32535700
H	5.58910200	−1.48194600	0.44095600
H	1.09941000	−1.64032600	−1.61345700
H	−0.48522500	−2.70183900	0.78904600
H	0.05735900	−3.65923800	−0.59195800
H	−1.13401700	−2.37606000	−0.82646200
H	−3.17671200	0.19214200	−1.53301500
H	−1.37297000	2.51791000	−0.26304000
H	0.00030500	4.61872000	−0.65842300
H	0.26628500	4.54185200	1.07922200
H	1.65435400	4.65893200	−0.01515500
H	2.02556700	−2.35111800	1.20886700
H	2.35224700	−3.29235400	−0.23982500
H	−1.68718900	−0.62054600	1.02614100
H	−1.48752600	1.30607500	−2.28350700
H	0.27055900	1.27587500	−2.33920000
H	−0.64748100	−0.23416100	−2.50660700
H	−5.02573500	−2.01467700	1.65081800
H	−3.30118700	−2.37324100	1.36044600
H	−3.75928100	−0.86852000	2.16555800
H	−1.92619700	1.56717900	1.91197000
H	−0.83738100	2.93223200	2.15913100
H	−0.21143700	1.29009300	2.29098700

Conformer 2

0 1			
C	-1.88100200	2.05367700	0.29306900
C	-0.74198100	2.69756000	0.02564500
C	-2.01238600	0.56789000	0.45519500
C	-3.26836200	0.02004800	-0.24099500
C	-3.50083200	-1.45059900	0.11977100
C	-4.71145700	-2.03416400	-0.61238100
C	-0.94890100	-1.69659300	0.48949300
C	0.20992500	-2.65809600	0.20072700
C	-0.76133400	-0.22893600	0.01589600
H	-0.75762900	-0.25460800	-1.08415600
O	3.94632000	-1.42289300	-1.39814400
C	4.09036600	-0.94625500	-0.28055700
C	2.98261000	-0.27827900	0.44999500
C	0.54749400	0.51489100	0.43803000
C	0.54816100	1.94541500	-0.22919200
C	-0.67246600	4.20075000	-0.04638800
C	-2.23151000	-2.26646300	-0.15231400
C	1.76660800	-0.18147100	-0.10746600
C	0.62728800	0.67898000	1.96479000
H	-2.15905400	0.37837500	1.53327400
C	5.42469200	-1.00982300	0.44111400
C	0.85827200	1.94204700	-1.74211000
H	-2.79177400	2.63046700	0.45931500
H	-3.15601000	0.12572900	-1.33009400
H	-4.14381500	0.61953000	0.04315300
H	-3.70019800	-1.49448500	1.20221300
H	-5.62216300	-1.46492700	-0.39173400
H	-4.55771600	-2.01041200	-1.69869400
H	-4.88701500	-3.07714700	-0.32359600
H	-1.10066500	-1.67626100	1.57927400
H	0.45770000	-2.68204700	-0.86705500
H	-0.07565300	-3.67566600	0.49308100
H	1.11886500	-2.39947200	0.74652300
H	3.19736800	0.10878300	1.44223200
H	1.36613900	2.49684400	0.25734700
H	0.01207100	4.59609600	0.71686100
H	-0.29360900	4.54990200	-1.01543400
H	-1.65602300	4.65374200	0.11212800
H	-2.07418000	-2.32808300	-1.24071200
H	-2.37903400	-3.29816300	0.19348500
H	1.67276700	-0.60954500	-1.10345000
H	1.51953400	1.24469500	2.25157900
H	-0.23748100	1.22628900	2.34574900

H	0.67106000	−0.29367600	2.46515900
H	6.16383900	−1.51222000	−0.18515300
H	5.77432600	0.00103700	0.68695200
H	5.31813200	−1.54941000	1.39091500
H	1.86661800	1.57281900	−1.94910200
H	0.78922900	2.95712900	−2.14469100
H	0.14270300	1.32435400	−2.29414900

Table S3 Comparison of ^{1}H and ^{13}C NMR data of compound **2** between the experimental values and literature values.

**Position**	**δ_C, type**		**δ_H (J in Hz)**	
	**reference (DMSO-d_6)**	**EXP (DMSO-d_6)**	**reference (DMSO-d_6)**	**EXP (DMSO-d_6)**
1	27.07, CH_2	27.04, CH_2	2.90(app. Bs)	2.97, (app. Bs)
2	22.10, CH_2	22.04, CH_2	1.80-1.89, m	1.86–1.91, m
3	22.12, CH_2	22.05, CH_2		
4	33.66, CH_2	33.59, CH_2	2.99 (app. Bs)	3.04, (app. Bs)
5	159.8, C	159.51, C		
6	146.28, C	146.19, C		
7	128.66, CH	128.58, CH	7.91, dd (0.7, 8.5)	7.94, d (8.4)
8	128.75, CH	128.79, CH	7.72, ddd (1.3, 7.0, 8.4)	7.75, m
9	127.03, CH	127.07, CH	7.61, ddd (1.3, 7.0, 8.4)	7.65, m
10	123.23, CH	123.21, CH	8.07, dd(1.0, 8.5)	8.12, d (8.4)
11	124.62, C	124.56, C		
12	140.02, C	140.01, C		
13	129.59, C	129.63, C		

Table.S4 Primer sequences used in the study.

Gene name	Forward Primer (5' - 3')	Reverse Primer (5' - 3')
cfos	F:TTACCCGCTCAACCAGACTC	R:TGACAGTTGGCACGAAAGAG
gria1b	F:TGTCCACAAA TCCCTCCGAAT	R:ATTCGCCATGGGTATGCCAG
gat1	F:ATGCTGTTTATCCTGTTCATCCG	R:TGTTGAAGGGGTTGTAGCTCC
ef1α	F:GCTCAAACATGGGCTGGTTC	R:AGGGCATCAAGAAGAGTAGTACCG
